# Building trust in clinical research: a systems approach to ethical engagement and sustainable outcomes

**DOI:** 10.3389/fphar.2025.1570899

**Published:** 2025-07-16

**Authors:** Johanna M. C. Blom, Veronica Rivi, Fabio Tascedda, Luca Pani

**Affiliations:** ^1^ Department of Biomedical, Metabolic and Neural Sciences, University of Modena and Reggio Emilia, Modena, Italy; ^2^ Center for Neuroscience and Neurotechnology, University of Modena and Reggio Emilia, Modena, Italy; ^3^ Department of Life Sciences, University of Modena and Reggio Emilia, Modena, Italy; ^4^ Consorzio Inter-Universitario per le Biotecnologie, Trieste, Italy; ^5^ Department of Psychiatry and Behavioral Sciences, University of Miami, Miami, FL, United States

**Keywords:** clinical research, engagement, empowerment, respect, transparency, autonomy, social alliance, emergence

## Abstract

Trust and trustworthiness are critical to the success of clinical research, profoundly influencing participant engagement, data integrity, and study outcomes. These behaviors emerge from complex, dynamic interactions within the clinical research ecosystem, involving stakeholders such as sponsors, participants, clinicians, researchers, and regulatory bodies. The rapid development of COVID-19 vaccines has underscored the potential of scientific advancements to build public trust in the scientific outcomes, while also exposing vulnerabilities in the procedural trust framework due to misinformation and historical unethical practices. This paper explores trust and trustworthiness as emergent properties within the complex systems of clinical research, highlighting their evolution through transparent communication, participant empowerment, and ethical governance. A systems approach is emphasized, where trust develops holistically, influenced by regulatory frameworks, interpersonal relationships, and the overall research environment. Practical implications include the adoption of adaptive consent models, interdisciplinary collaboration, and the integration of continuous feedback mechanisms. To address trust erosion, especially among marginalized communities, we advocate for participatory research approaches and the development of new professional competencies, such as the role of a Clinical Research Liaison. This role would ensure ongoing alignment with community needs, enhance transparency, and maintain ethical standards, ultimately fostering a research environment where trust and trustworthiness thrive, benefiting both participants and the broader scientific community. A roadmap for future efforts includes the systematic incorporation of these elements into clinical research practices to enhance trust and improve research outcomes.

## Introduction

This paper presents a systems approach to fostering trust in clinical research through tailored strategies at multiple levels. Trust and trustworthiness are essential to the success of clinical research, influencing participant engagement, data integrity, and overall study outcomes. These behaviors emerge from complex interactions among key stakeholders, such as, sponsors, participants, clinicians, researchers, and regulatory bodies. Here, we examine trust as a multi-layered and emergent property within clinical research, proposing strategies for building it at systemic and interpersonal levels. Recent advancements, including the rapid development of COVID-19 vaccines, have showcased the potential of scientific breakthroughs to strengthen public trust in research (Polack et al., 2020; Baden et al., 2021). However, this same period has underscored the vulnerability of trust (Schiavo, 2022; Schiavo and Chou, 2023), particularly when confronted with widespread misinformation (Wang et al., 2023; Furman, 2023) and historical instances of unethical research, such as the Tuskegee Syphilis Study and the Surgisphere scandal (Jones, 1981; Ledford et al., 2020). These challenges highlight the importance of transparency, ethical standards, and effective communication in maintaining public confidence in medical science (O’Doherty, 2022; Hawley, 2014). They also cast long shadows over public perception of clinical research, emphasizing the importance of understanding how trust and trustworthiness develop and manifest in clinical settings. Recent data from the Center for Information and Study on Clinical Research Participation (CISCRP) show that while trust in the conduct and governance of clinical research (e.g., processes, ethical oversight) and trust of its outcomes (e.g., reliability and applicability of findings) have both improved, significant gaps remain, with only about 52% of the general public expressing trust in clinical research (Center for Information and Study on Clinical Research Participation CISCRP, 2020)*.* Here we explore trust as an emergent property in clinical research, contextualizing its significance amid recent public health challenges, such as the COVID-19 vaccine trials. These events underscore the dual importance of rapid, transparent communication and the obstacles that arise when addressing public trust. By examining trust as an outcome of complex, interdependent systems, this article highlights the need for a strategic approach to build and maintain trust across the clinical research ecosystem.

### The importance of trust in clinical research: ensuring transparency in processes and credibility of outcomes

Trust is foundational to the success of clinical research, influencing not only participation and retention but also the integrity of the data collected and the broader perception of science. Therefore, we distinguish “trust in clinical research,” referring to confidence in the ethical, procedural, and operational integrity of research processes, from “trust of clinical research,” referring to confidence in the reliability, relevance, and societal value of its outcomes but also participant trust, emerging through direct engagement in the research process, and public trust, formed through broader social narratives and institutional reputation.

More importantly, trust is not a static or singular construct—it is multifaceted, dynamic, and shaped by interpersonal, institutional, and systemic factors. Several core elements contribute to the cultivation of trust in clinical research, transparency, respect, autonomy, and empowerment.

Transparency ensures that patients are well-informed about research aims, procedures, treatments and associated risks and benefits, enabling informed and ethical decision-making (Staunton et al., 2024). Respect affirms participants' values, dignity and perspectives supporting empathetic engagement and shared decision-making. Autonomy reinforces individuals’ rights to choose their healthcare paths, and make voluntary and informed choices enhancing trust through active participation (O’Neill, 2002). Empowerment, on the other hand, equips them with the knowledge and confidence to engage meaningfully in their care, reinforcing trust in the reliability and credibility of the information provided (Kennedy et al., 2013).

In healthcare, where uncertainty and vulnerability are often inherent, these elements acquire heightened relevance. Patients frequently look to healthcare professionals not only for treatment but also for reassurance, interpretive guidance, and decision support (Shea et al., 2009). In such moments, the concept of epistemic safety becomes critical. Epistemic safety refers to the patient’s belief that their healthcare providers are not only competent but also acting reliably and in good faith. It fosters confidence that diagnoses, treatments, and communications are well-founded and trustworthy (Cook et al., 2013; Andersen, 2024; Osterberg and Blaschke, 2005). Closely related is the concept of epistemic trust, or the willingness to accept information from others as trustworthy, relevant, and motivated by goodwill. This is particularly important in clinical research, where participants may lack the technical expertise to independently evaluate, often complicated, study protocols (Cook et al., 2013; Dormandy and Dormandy, 2020; Lalumera, 2024). This reliance is amplified by the human tendency toward cognitive closure, a psychological drive to resolve ambiguity, which further reinforces the importance of trustworthy communication (Dormandy and Dormandy, 2020). Higher levels of trust correlate strongly with positive outcomes, such as better treatment adherence, more proactive engagement, and increased willingness to participate in clinical trials (Mitchell and Newman, 2002). Conversely, when trust is compromised, whether by past un-ethical practices, misinformation, or unclear communication, participants may disengage, withdraw, or decline to participate in clinical research, jeopardizing both the quality and ethics of research (Sadoff et al., 2021).

While this paper does not propose a definitive model of trust, existing interdisciplinary frameworks help clarify how trust functions within clinical research ecosystems. One such framework—drawn from neuroscience, psychology, and behavioral economics—identifies five interrelated dimensions that support interpersonal trust: trustworthiness, responsibility, understanding, shared goals, and transparency (Krueger and Meyer-Lindenberg, 2019). Trustworthiness refers to the perceived competence, integrity, and consistency of researchers and clinicians, an essential foundation for establishing epistemic trust. Responsibility emphasizes the ethical obligations of institutions and professionals to safeguard participants wellbeing, privacy, and rights. Understanding reflects the need for mutual comprehension and empathetic communication, ensuring that participants are not only informed but also supported in navigating uncertainty and complex information. Shared goals recognize the alignment of individual participation with broader public health objectives, reinforcing a sense of solidarity and collective benefit.

Finally, as indicated earlier, transparency ensures ongoing clarity around research intentions, risks, data practices, and institutional accountability. While these elements are not presented as an explicit framework within this article, they offer a useful conceptual backdrop that helps to elucidate the layered nature of trust as it is explored in the work that follows. Ultimately, trust in clinical research is emergent and multi-layered. It arises from interpersonal interactions, team dynamics, institutional policies, and the broader socio-political environment. Recognizing and responding to this complexity is crucial. When trust is actively nurtured through transparent processes, respectful relationships, participant empowerment, and shared goals, the clinical research enterprise becomes not only more ethical but also more inclusive, resilient, and effective.

### Emergence and trust in clinical research

Emergence, a concept where complex systems and patterns arise from relatively simple interactions, is particularly relevant to understanding trust and trustworthiness in clinical research (Wolff and Larson, 2010; Occa et al., 2024). Trust emerges from the dynamic interplay among various stakeholders within the research ecosystem (Gutnick et al., 2024) and engages various levels of complexity:

1. Individual Trust: Established in direct researcher-participant interactions, built on transparency and respect
2. Team-Level Trust: Reinforced through cohesive, cross-functional teams that consistently adhere to ethical standards
3. Organizational Trust: Achieved by research institutions through consistent practices and transparent communication
4. System-Level Trust: A broader trust framework encompassing the clinical research ecosystem, fostered through standardized practices, accountability, and cross-sector collaboration

Therefore, in clinical research, trust develops across four distinct layers—individual, team, organizational, and system—each presenting unique challenges and requiring specific strategies.

At the individual level, trust depends on clear and transparent communication. Participants may struggle to fully understand complex research protocols, especially when historical unethical practices have fostered distrust. Adaptive consent models, like those in the 100,000 Genomes Project (England, 2025), allow participants to make informed decisions on an ongoing basis, strengthening trust by empowering them with control over their data. At the team level, trust requires balancing data privacy, transparency, and ethical standards. Diverse expertise and roles can lead to friction, particularly in data-sensitive environments (Gutnick et al., 2024). Establishing data privacy protocols, such as provided by the EU General Data Protection Regulation (GDPR) (Chico, 2018) and regular team feedback sessions foster mutual accountability and ensures that ethical standards are consistently applied by the team members (EMA, 2025). At the organizational level, trust is built by aligning ethical responsibilities with operational priorities.

**FIGURE 1:**
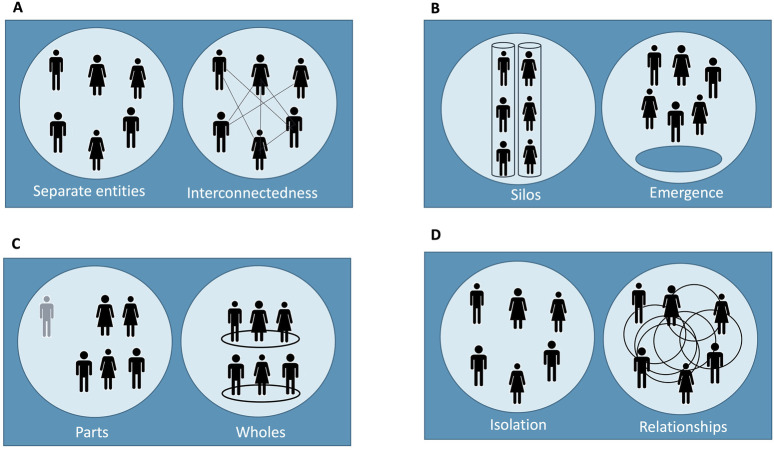
Emergent model of trust in clinical research across four layers. This figure illustrates the dynamic nature of trust as it develops across four distinct layers within the clinical research ecosystem: **(A)** individual, **(B)** team, **(C)** organizational, and **(D)** system level. Each panel represents a trust layer and depicts two contrasting states of relational structure: disconnected/isolation versus interconnected/emergent interaction. Each pair of diagrams is intended to contrast a **minimal or** absent trust scenario (left) with an emergent trust scenario (right), reflecting how trust can be either inhibited or fostered depending on the structure and interaction within and across layers. This design emphasizes that trust is not a static feature but an evolving property shaped by relational dynamics, ethical coherence, and systemic integration—aligned with the systems-thinking approach outlined in the text: **(A)** Individual Trust: Depicts the contrast between separate, transactional interactions *versus* engaged, respectful researcher-participant relationships where mutual understanding is present; **(B)** Team-Level Trust: Shows the difference between siloed or compartmentalized team members working in isolation versus cohesive, interdisciplinary collaboration where roles are aligned through shared ethical standards; **(C)** Organizational Trust: Highlights fragmented parts functioning independently versus a unified organization where trust emerges from transparent policies and consistent internal communication across departments; **(D)** System-Level Trust: Contrasts isolated actors operating without coordination versus a networked system where trust emerges from standardized governance, shared accountability, and inclusive partnerships across the clinical research ecosystem.

Misconduct or lack of transparency can significantly impact public perception and the public’s trust in the organization (Chico, 2018). To counter this, transparent policies on data use, participant rights, and appointing clear roles help align institutional actions with participant expectations, enhancing organizational trust (EMA, 2025). Lastly, at the system level, trust requires alignment among diverse stakeholders and addressing historical mistrust, especially within marginalized communities (Gadsby, 2023). By adhering to ethical governance frameworks and incorporating participatory research models, the research ecosystem can create an inclusive environment that fosters trust across all levels. Overall, building trust in clinical research is a complex multi-layered process that begins with individual interactions and extends to system-wide practices. Recognizing trust as an emergent property highlights the need for tailored approaches to transparency, ethics, and collaboration at each level. This perspective sets the stage to explore trust from different angles (see Figure 1) and helps us understand how trust can be developed and sustained throughout the research ecosystem.

### Trust from a systems approach

A key characteristic of a systems approach is radical novelty, wherein each level of complexity within the clinical research ecosystem gives rise to emergent properties and dynamic interactions that were not present at preceding levels. These emergent behaviors stem from the interconnectedness of system components, requiring a holistic perspective to understand and navigate their implications. For instance, transitioning from individual patient-doctor interactions, where trust is primarily built through direct, personalized communication and shared decision-making, to a full-scale clinical trial, introduces new challenges and opportunities for trust-building that are not present in the initial stages, which typically involve early engagement efforts, community-based outreach, and transparent information-sharing to address concerns and foster participation (Gutnick et al., 2024). Different types of clinical research demand different kinds of trust. In interventional clinical trials, participants often face acute vulnerability due to experimental treatments, invasive procedures, or the urgency of health conditions. These trials require high levels of epistemic and interpersonal trust—participants must believe in the competence and integrity of investigators and feel secure that risks are ethically justified and transparently conveyed. Conversely, population-based observational cohorts typically involve long-term participation with lower physical risk but raise complex questions about data use, privacy, and long-term governance. Here, institutional and systemic trust, grounded in transparent data stewardship and sustained engagement, is critical. Recognizing these differences allows researchers to tailor trust-building strategies to study context. For instance, adaptive consent may be prioritized in observational studies, whereas interventional trials may benefit from the presence of a dedicated professional figure to support informed decision-making under conditions of stress or uncertainty. Explicitly aligning trust strategies with study type ensures more ethical and effective participant engagement.

The multifaceted and evolving nature of trust is illustrated by the COVID-19 vaccine trials. Initially, individual patient consultations about the vaccine involved personalized communication and trust-building. As these consultations scaled up to massive public vaccination campaigns, new layers of trust had to be developed, involving transparent communication of trial results, messaging to the general public, and addressing vaccine hesitancy (Pisani and Maarten, 2017; Mahmood et al., 2014). Furthermore, coherence within the research framework enables trust to establish a stable network of interactions among stakeholders, enhancing the overall reliability of clinical research processes (Gadsby, 2023). A notable example of this is the long-running Framingham Heart Study, where consistent communication and engagement with participants over decades have built a stable foundation of trust and long-term participant retention (Mahmood et al., 2014). Thus, trust and trustworthiness, viewed as holistic properties of the entire clinical research system, cannot be fully understood by examining individual components in isolation. Instead, they require a comprehensive view that considers the entire research environment and process (Pisani and Maarten, 2017). The entirety of the clinical research environment, including ethical considerations, regulatory frameworks, and interpersonal relationships, contributes to a comprehensive building of trust.

The MRCT Center’s efforts to harmonize ethical standards across global trials exemplify this approach, ensuring that trust is created and maintained universally (Center MRCT, 2020).

Within this context, the dynamic nature of trust and trustworthiness emphasizes their continuous evolution, shaped by ongoing interactions, feedback, and changes within the environment, such as advancements in ethical standards and changes in regulatory requirements (Staunton et al., 2024). For instance, new guidelines on data privacy like the GDPR in the EU significantly impact how trust is perceived and maintained among participants (Chico, 2018). Another example is the dynamic consent model in biobanking, which allows participants to continually update their consent preferences, thus maintaining control and consequently trust through active engagement (Staunton et al., 2024). Finally, downward causation, where the system of clinical research influences the behavior of its individual components, underscores the importance of robust ethical guidelines and regulatory oversight (Kaye et al., 2015; Budin-Ljøsne et al., 2017). For example, the implementation of adaptive consent processes in clinical trials demonstrates how institutional practices can adapt to enhance trust and engagement through continuous feedback and participant interaction. Historical examples, such as the Nurses' Health Study, the Framingham Heart Study and the UK Biobank’s implementation of dynamic consent models, illustrate the concept of emergence in trust-building (Mahmood et al., 2014; Fleming et al., 2020; Greenhalgh and Wieringa, 2011). These initiatives demonstrate how ongoing communication, participant empowerment, and adaptability can enhance trust, ensuring long-term engagement and adherence to ethical research practices.

### Trust and argumentation in clinical research

Trust and trustworthiness are fundamental for the acceptance and impact of argumentation in clinical research (Walton, 2007; Habermas, 1984). Trust involves belief in the arguer’s competence and sincerity, while trustworthiness is rooted in the arguer’s expertise and integrity. Without these qualities, even logically sound and well-founded arguments may be dismissed (Walton, 2007; Habermas, 1984). Effective argumentation in clinical research requires transparent communication of methodologies and findings, coupled with proactive efforts to address potential concerns and misconceptions. Researchers who demonstrate ethical commitment and transparency are more likely to gain trust from participants and the public, ensuring that their arguments are perceived as both knowledgeable and ethically grounded (Simon et al., 2012). Transparent and ethical argumentation has successfully built trust in various clinical research settings. One notable example is the All of Us Research Program, an initiative by the National Institutes of Health (NIH) (National Institutes of Health NIH, 2018). This program emphasizes transparent communication and participant engagement, ensuring that individuals are well-informed about the research goals, procedures, and their rights. This openness has established the program’s reputation for trustworthiness and garnered substantial public support (National Institutes of Health NIH, 2018).

Similarly, institutions like the Mayo Clinic have set high standards for ethical practices a transparent communication, including detailed consent processes, ongoing participant education, and regular reporting of study results, which foster trust through consistent and clear communication (Clinic, 2024).

### Practical applications and case studies

#### Adaptive consent models

Adaptive consent models represent a transformative approach to participant engagement in clinical research, moving beyond static, one-time agreements to enable ongoing dialogue and decision-making. Unlike traditional consent methods, which often fail to accommodate changing participant preferences and may contribute to disengagement, adaptive consent promotes sustained trust by allowing individuals to update or withdraw their consent as new information or circumstances emerge (Budin-Ljøsne et al., 2017). This flexibility is exemplified in initiatives such as the 100,000 Genomes Project, where dynamic consent platforms support participant autonomy and transparency by enabling people to revisit and revise their consent decisions throughout the research lifecycle (England, 2025).

Importantly, adaptive consent does not stand alone. It aligns closely with participatory research practices, such as community-based participatory research (CBPR) and co-design methodologies, which further deepen engagement by embedding participants in all stages of the research process. CBPR involves community members as equal partners in planning, data collection, and dissemination, ensuring that study goals are responsive to local needs. Similarly, co-design methods like those employed in the All of Us Research Program (National Institutes of Health NIH, 2018) empower participants to shape study design and decision-making, enhancing inclusivity and cultural relevance. Together, adaptive consent and participatory frameworks signal a broader shift toward a participant-centered research paradigm. They embody transparency, foster mutual respect, and promote long-term engagement—all of which are central to sustaining trust and trustworthiness in clinical research. These approaches also carry significant implications for emerging ethical priorities, such as the return of data to participants and compliance with data protection regulations like the GDPR. To address these evolving demands, research institutions must rethink consent protocols to ensure clarity on how data are used, stored, and shared, while developing governance structures that uphold participant rights (Cohen and Mello, 2019). Viewed through a systems lens, adaptive consent plays a pivotal role across the four layers of trust within clinical research. At the individual level, it reinforces epistemic trust and epistemic safety, giving participants a sense of control and confidence in their involvement (Kaye et al., 2015; Budin-Ljøsne et al., 2017). At the team level, adaptive consent demands collaborative protocols and communication across disciplines—such as ethics, clinical care, and data management—fostering shared responsibility and ethical coherence (EMA, 2025; Muller et al., 2023).

At the organizational level, the adoption of dynamic consent reflects a visible commitment to transparency and participant engagement, strengthening institutional credibility and trustworthiness (Chico, 2018; Staunton et al., 2024). Finally, at the system level, the integration of adaptive consent into regulatory and ethical standards supports the harmonization of governance and fosters public trust across institutions and jurisdictions (Chico, 2018; Cohen and Mello, 2019).

#### Participatory approaches in clinical research

Adopting participatory research frameworks is essential for fostering trust and enhancing the relevance, inclusivity, and long-term impact of clinical studies. These approaches prioritize meaningful engagement with individuals who have lived experience, ensuring their insights directly shape research design, implementation, and outcomes. Historically, trust in clinical research has been undermined by unethical practices, especially in marginalized communities. Participatory models address this legacy by affirming values such as transparency, autonomy, and ethical conduct (Kennedy et al., 2013; Kolber, 2006). By actively involving participants in study development—from planning and data collection to dissemination—these methods demonstrate a sustained commitment to participant welfare, cultural sensitivity, and shared ownership. Programs like the All of Us Research Program exemplify the power of participatory models to improve trust and engagement through co-design and inclusive governance structures (National Institutes of Health NIH, 2018; Denny et al., 2019). Similarly, the Bridging Research, Accurate Information, and Dialogue (BRAID) model promotes continuous, bidirectional communication with community members, using tools such as Conversation Circles to elicit feedback and tailor research strategies (Simons et al., 2017). These approaches not only build trust but also create safe spaces for community expression, fostering a more responsive and resilient research environment. Participatory designs also support long-term engagement and knowledge translation by making studies more relevant to community needs, which in turn enhances the credibility and ethical robustness of the research (Cook et al., 2013; Simons et al., 2017). Importantly, the impact of participatory research spans across the four layers of trust. At the individual level, participants become co-creators rather than passive subjects, fostering a sense of agency and recognition (Kennedy et al., 2013; Simons et al., 2017). At the team level, the integration of community advocates and diverse stakeholders enhances reflexivity, cultural competence, and interpersonal trust among researchers and collaborators (Gutnick et al., 2024; Kolber, 2006). At the organizational level, institutions that embed mechanisms such as advisory boards, co-leadership models, and open dissemination practices signal a long-term commitment to equity and responsiveness (Clinic, 2024; Wallerstein et al., 2019). Finally, at the system level, participatory research supports the democratization of science, shifting power toward communities historically excluded from decision-making and strengthening public trust in research institutions (Blom et al., 2023; Richardson et al., 2017). Numerous case studies reinforce these dynamics. For example, Mittal and Gera demonstrated how incorporating adaptive consent with community input enhanced participant control and trust (Wallerstein et al., 2019; Muller et al., 2023; Kolbert, 2017).

The Mayo Clinic’s use of community advisory boards has similarly been lauded for promoting transparency and aligning research with patient needs (Clinic, 2024). Furthermore, community-based participatory research (CBPR) initiatives have proven effective in increasing trust and collaboration among minority populations, resulting in more culturally attuned and socially responsive research outcomes (Simon et al., 2012; Wallerstein et al., 2019; Muller et al., 2023; Haroutounian at al., 2024; Ballantyne and Schaefer, 2018). Collectively, these examples underscore that participatory approaches are not only ethically compelling but also strategically essential for building sustainable trust in clinical research.

#### Solidarity and collective responsibility

The ethical principle of *solidarity* in clinical research underscores a commitment to collective wellbeing, emphasizing that participants' contributions serve not only their personal interests but also benefit broader public health. This principle fosters mutual support and shared responsibility, particularly crucial in addressing health challenges that require collective responses, such as infectious disease outbreaks or health disparities. Solidarity-based approaches encourage participants to engage meaningfully in research (Goold and Lipkin, 1999) recognizing that their involvement aids others who face similar health vulnerabilities (Cook et al., 2013; Blom et al., 2023). One impactful example of solidarity in action is community health partnerships, where researchers collaborate with local communities to address specific health needs. During the Ebola outbreak in West Africa, for instance, researchers worked with community leaders to build trust and implement public health measures that respected local customs (Richardson et al., 2017). By engaging with communities and valuing their input, researchers fostered a sense of solidarity, aligning public health goals with community values and thereby enhancing health outcomes. Informed consent models that embrace solidarity further strengthen this ethical foundation. For example, dynamic consent can be seen as a reflection of informational solidarity, ensuring that participants are empowered with ongoing control over their data while still contributing to the greater good of the research community (Wiertz, 2023; Wiertz and Boldt, 2022; Samuel et al., 2017). Additionally, broad consent models can align with solidarity by supporting the broader goals of health data research, allowing participants to opt into a wider range of studies that address shared health concerns. In this way, solidarity-based consent models not only respect individual autonomy but also emphasize the collective benefits that emerge when participants feel they are contributing to a just cause, ultimately enhancing both participation and trust (O’Doherty et al., 2021).

### Developing professional competencies for trust in clinical research

Addressing the complexities of trust in clinical research requires developing specialized professional competencies that prioritize patient-centered care. This involves a proactive approach that not only manages the inherent challenges of research but also strengthens communication and participant engagement, key factors for improving both enrollment and retention.

Emerging roles, such as the “Clinical Research Liaison” (CRL) are essential in this regard, requiring a blend of communication, research, and organizational skills to ensure that participants’ needs and concerns remain central throughout the study process (Table 1). These professionals simplify complex research protocols, manage informed consent, and promote transparent interactions among stakeholders. The Clinical Research Liaison serves as a bridge across different layers of trust within clinical research, reinforcing trust holistically. At the individual level, this role fosters transparency and respect by ensuring participants understand complex protocols and their rights, directly supporting informed consent through clear, accessible communication. At the team level, the Clinical Research Liaison upholds ethical standards by promoting GDPR compliance and data privacy protocols, fostering accountability and cohesive practices within cross-functional teams. At the organizational level, the Clinical Research Liaison enhances transparency by aligning institutional actions with participant expectations, ensuring that policies on data use and participant rights reflect ethical commitments. Finally, at the systems level, this role builds trust by facilitating cross-sector collaboration and supporting standardized governance practices, addressing historical mistrust and creating a trustworthy, inclusive research ecosystem. Clinical trials are becoming increasingly complex, involving more diverse patient populations, stringent regulatory requirements, and heightened ethical considerations. As a result, there is a pressing demand for a role that not only ensures compliance and efficiency but also prioritizes patient engagement, ethical transparency, and trust-building throughout the research process.

**TABLE 1:** Key competencies for the clinical research liaison.

Competency	Description
Communication skills	Ability to clearly convey complex information, address participant questions, and ensure understanding among stakeholders, including physicians, trial monitors, and patients
Ethical knowledge	Familiarity with ethical standards, regulations, and protocols in clinical research, ensuring all communications and practices comply with standards such as GCP (Good Clinical Practice) and GDPR
Interpersonal skills	Empathetic, responsive engagement with participants based on respect, building trust by addressing individual needs and fostering an open, supportive trial environment
Data management	Proficiency in securely managing data while adhering to privacy standards like GDPR, ensuring participants' information remains protected throughout the trial
Practical problem-solving skills	Ability to address daily challenges in clinical trial management promptly and effectively, balancing the needs of participants, researchers, and regulatory bodies
Rigor of clinical research	Commitment to upholding scientific integrity while prioritizing participant wellbeing, ensuring the trial meets high research standards while remaining patient-centric

By placing trust and ethical transparency at the core of clinical research, this role not only enhances immediate trial outcomes but also reinforces the broader societal value of research, helping to build public confidence in clinical trials and increase participation among historically underrepresented populations. Unlike traditional roles such as clinical trial managers or study coordinators, whose responsibilities center primarily on regulatory compliance, scheduling, data integrity, and logistical operations, the Clinical Research Liaison (CRL) centers their work on ethical communication, participant advocacy, and relational continuity throughout the study. As highlighted by the competencies in Table 1, the CRL is uniquely positioned to translate complex protocols into accessible information, support adaptive consent practices, and maintain open channels for feedback and dialogue. While many of these functions may be carried out informally by existing staff, the formalization of this role acknowledges the growing ethical and interpersonal demands placed on research teams. Although overlaps exist, particularly in informed consent and participant interactions, the CRL role formalizes the interpersonal and trust-building responsibilities that are often unevenly distributed or informally handled by other staff. This delineation is intended to enhance the clarity of responsibilities, reduce role ambiguity, and strengthen participant support without disrupting existing operational workflows. Depending on institutional resources and trial design, the CRL role could be implemented as a standalone position or integrated into existing roles with expanded training and adjusted responsibilities.

For example, in smaller studies, a trained coordinator could adopt core CRL functions, whereas in large or high-risk trials, particularly those involving vulnerable populations, a dedicated CRL may be essential. This flexibility enables institutions to tailor implementation without wholesale restructuring of their staffing models.

Cost considerations are understandably central to any discussion of workforce expansion. While the introduction of a new role may initially increase personnel costs, we contend that the long-term return on investment justifies this expenditure. Nonetheless, budget constraints remain a significant barrier, particularly for publicly funded or resource-limited institutions. The implementation of the CRL role may require upfront investment in hiring, training, and integration. However, potential cost offsets include improved participant enrollment and retention, reduced protocol deviations, and fewer ethical violations, all of which can translate into financial savings through smoother trial execution and fewer regulatory delays. Furthermore, in trials with historically low enrollment from marginalized populations, a CRL can help achieve diversity goals, avoiding costly recruitment shortfalls and increasing external validity. Institutions may also consider phased implementation or shared CRL responsibilities across multiple trials to optimize budget alignment.

Moreover, by proactively addressing participant concerns and minimizing misunderstanding or dissatisfaction, the CRL can reduce the administrative and reputational burdens associated with disengagement or complaints. Importantly, the CRL also supports broader societal objectives: “By placing trust and ethical transparency at the core of clinical research, this role not only enhances immediate trial outcomes but also reinforces the broader societal value of research, helping to build public confidence in clinical trials and increase participation among historically underrepresented populations” (personal communication)”. This alignment with inclusion and justice is particularly critical in addressing legacies of structural mistrust and ensuring that research outcomes reflect diverse communities (Smirnoff et al., 2018; Sonstein et al., 2024).

### Piloting and evaluation

To translate the proposed frameworks into practice, the next phase should involve structured piloting and empirical evaluation. Priority efforts include pilot testing the CRL role in diverse clinical settings to assess its impact on informed consent quality, participant retention, and ethical communication (Kraft et al., 2018). Mixed-methods evaluations, including participant satisfaction surveys and trust metrics, can help measure effectiveness (Damschroder et al., 2009). Deploying adaptive consent platforms across both interventional and observational trials, with attention to usability, comprehension, and consent revision rates (Beskow and Weinfurt, 2019). Evaluations should track participant autonomy and long-term engagement. Embedding participatory research practices, such as community advisory boards, co-design methods, and culturally tailored outreach, into existing research infrastructures (Wallerstein and Duran, 2010). Outcomes to monitor include enrollment diversity, protocol adherence, and community trust indicators. Cross-institutional collaboration is recommended to standardize evaluation metrics and share implementation findings, ensuring broad applicability and refinement across settings. These actions will help operationalize the manuscript’s systems-thinking framework, enabling data-driven refinement and sustainable integration of trust-enhancing practices across the clinical research enterprise.

## Conclusion and future directions

This manuscript has outlined a systems-based approach to trust in clinical research—one that integrates professional competencies, ethical infrastructures, adaptive consent models, and participatory frameworks. Trust, in this context, is not a static endpoint but a dynamic, evolving principle shaped by the interactions among participants, researchers, institutions, and broader sociotechnical ecosystems. However, trust in clinical research faces new and evolving threats. The rise of digital misinformation, amplified by social media, has fueled public skepticism. To counter this, future strategies must extend beyond ethical compliance to include digital literacy, transparent communication, and partnerships with trusted community voices. These efforts are critical to ensuring that research is not only ethically sound but also socially legible and contextually relevant. Furthermore, trust-building is not one-size-fits-all. Interventional trials demand immediate, high-stakes trust due to inherent risks, whereas observational studies raise long-term concerns around data use and representation. A systems-oriented framework must therefore offer tailored strategies responsive to study type, participant vulnerability, and degree of involvement. To advance this agenda, future work should focus on structured piloting and evaluation of key innovations. Priority actions include testing the CTR role as a bridge between participants and research teams; developing digital, adaptive consent tools that support comprehension and autonomy; and embedding co-design and participatory research practices across diverse settings. Evaluation should incorporate both qualitative and quantitative measures, capturing outcomes such as informed consent quality, retention, perceptions of fairness, and overall trust.

A second pillar of this framework is workforce development. Building on the Joint Task Force for Clinical Trial Competency, we advocate for expanding competency frameworks to include trust-building skills, such as cultural humility, advocacy, and power-sensitive communication. These skills are essential for cultivating an ethically grounded and globally competent research workforce. At the systems level, coordinated action among regulators, sponsors, and public health institutions is essential to prevent fragmented governance from undermining trust. Moreover, emerging technologies, such as blockchain for data transparency and AI-driven risk modeling, offer new opportunities but also new responsibilities. Their ethical adoption hinges on transparency, governance, and active engagement from all stakeholders. Specifically, aligning conceptual models of trust with their practical implementation, as emphasized in organizational trust research (McEvily and Tortoriello, 2011), ensures that innovative strategies remain grounded in rigorously defined, evidence-based principles.

Thirdly, we must consider the digital age, social media, and the erosion of trust. In the digital era, social networks serve as influential amplifiers of both accurate information and misinformation. Platforms such as Facebook, Twitter, TikTok, and YouTube have become powerful tools for shaping public attitudes toward clinical research (Ventola, 2014; Kanchan and Gaidhane, 2023). Unfortunately, they also provide fertile ground for the spread of conspiracy theories—such as claims that clinical trials are exploitative, that vaccines are harmful, or that pharmaceutical companies conceal data (Broniatowski et al., 2018). These narratives often tap into pre-existing distrust, especially among communities with historical reasons for skepticism (George et al., 2014). As a result, even scientifically sound and ethically conducted trials can face widespread public resistance.

To address this, clinical researchers must not only engage in rigorous science but also invest in digital communication strategies that are transparent, culturally relevant, and responsive to online misinformation. Partnering with community influencers, patient advocates, and digital health communicators can help bridge the gap between science and public perception (Chou et al., 2018; Larson, 2020). Ultimately, navigating this landscape requires recognizing that trust is no longer built solely through formal channels, but also in real-time, digital discourse.

Finally, future research must explore how trust manifests across geographies, demographics, and study types. Multidisciplinary inquiry, drawing from ethics, psychology, communication science, and health systems research, is needed to refine adaptable models of trust that reflect the complexity of today’s clinical research landscape.

By embracing continuous ethical innovation and systems thinking, clinical research can build a resilient foundation of trust, one that supports transparency, inclusivity, and scientific integrity. Trust, viewed not as a passive state but as a generative force, is essential to the future of ethically responsible and socially impactful clinical research.

## Data Availability

The original contributions presented in the study are included in the article/supplementary material, further inquiries can be directed to the corresponding author.
